# Recent Studies on Thermally Conductive 3D Aerogels/Foams with the Segregated Nanofiller Framework

**DOI:** 10.3390/polym14224796

**Published:** 2022-11-08

**Authors:** Mohammad Owais, Aleksei Shiverskii, Amit Kumar Pal, Biltu Mahato, Sergey G. Abaimov

**Affiliations:** 1 Center for Petroleum Science and Engineering, Skolkovo Institute of Science and Technology, 121205 Moscow, Russia; 2 Center for Energy Science & Technology, Skolkovo Institute of Science and Technology, 121205 Moscow, Russia

**Keywords:** thermal management applications, thermal interface materials, nanocomposite aerogels, thermal conductivity, graphene, graphene oxide, boron nitride

## Abstract

As technology advances toward ongoing circuit miniaturization and device size reduction followed by improved power density, heat dissipation is becoming a key challenge for electronic equipment. Heat accumulation can be prevented if the heat from electrical equipment is efficiently exported, ensuring a device’s lifespan and dependability and preventing otherwise possible mishaps or even explosions. Hence, thermal management applications, which include altering the role of aerogels from thermally insulative to thermally conductive, have recently been a hot topic for 3D-aerogel-based thermal interface materials. To completely comprehend three-dimensional (3D) networks, we categorized and comparatively analyzed aerogels based on carbon nanomaterials, namely fibers, nanotubes, graphene, and graphene oxide, which have capabilities that may be fused with boron nitride and impregnated for better thermal performance and mechanical stability by polymers, including epoxy, cellulose, and polydimethylsiloxane (PDMS). An alternative route is presented in the comparative analysis by carbonized cellulose. As a result, the development of structurally robust and stiff thermally conductive aerogels for electronic packaging has been predicted to increase polymer thermal management capabilities. The latest trends include the self-organization of an anisotropic structure on several hierarchical levels within a 3D framework. In this study, we highlight and analyze the recent advances in 3D-structured thermally conductive aerogels, their potential impact on the next generation of electronic components based on advanced nanocomposites, and their future prospects.

## 1. Introduction

While heat dissipation is becoming a critical concern for electronic devices, technology continues to develop toward constant downsizing and higher power density. Heat accumulation can be avoided if the heat from electrical equipment is efficiently exported, such that the electronic gadget’s lifetime or dependability is not compromised; otherwise, the device may cause accidents or perhaps even explode [1,2]. As a consequence, applications such as thermal management, which includes changing the role of aerogels from thermally insulative to thermally conductive, have recently been a hot topic for aerogels-based thermal management applications (TMAs) and thermal interface materials (TIMs). Polymers present ideal matrix materials due to their mechanical stability, flexibility, and high formability, but their thermal conductivity (TC) must be improved because they are thermal insulators. To do this, a highly thermally conductive filler is dispersed within the polymeric insulating matrix, to provide continuous routes through which heat can be carried across the material and then recovered or discharged. In addition, 2D nanofillers such as graphene and boron nitride have, in recent years, received a lot of interest because of their remarkable TC, which could give a significant increase in the composites’ TC [2,3,4,5]. Alternative routes include carbon nanotubes and cellulose carbonization. When fabricating such 3D structures, however, various important parameters, for 1D and 2D materials as nanofillers, must be highly considered in order to fabricate a thermally conductive functional materials for TMAs. For instance, the dimensional parameters of the flakes, the coherence of the structure, the contractual interactions between the flakes, and their purity [6].

Aerogels are perfect 3D interconnected designs with unique properties provided by tenuous networks of nanosheets or filaments; they are typically fabricated via sol–gel, freeze drying, and other phase-separating and drying techniques and possess remarkable properties, such as an extraordinarily high specific surface area, great flexibility, low density, variable tunable porosity, low dielectric constant, and low TC. Due to the abovementioned advantageous physical features, they present a large amount of promise for applications as adaptable absorbent materials [7] and for their uses in EM shielding [8], thermal insulation [9], and wearable pressure-sensing materials [10], to be employed as a multifunctional aerogel material. The latest trends show that the modification of these low thermally conductive 3D aerogels/foams into highly thermally conductive 3D structures with excellent electrical insulation has been a popular area of discussion, specifically in the field of thermal management in the electronics industry, as thermal interface materials or phase-change materials in recent years [11,12,13,14,15]. At present, several types of thermally conductive 3D aerogels frameworks are being focused on, including epoxy-infiltrated or cellulose-based composite aerogels, with carbonaceous and boron nitride nanomaterials and fibrous materials being currently most widely used as fillers. However, currently and in the near future, thermally conductive 2D materials such as Mxene [16,17,18], transition-metal dichalcogenide (TMDC)-like molybdenum disulfude MoS_2_ [19,20], could play a significant role in scientific breakthroughs, as 3D structures in applications related to TMA and TIMs. For instance, numerous attempts have been made to fabricate three-dimensional (3D) graphene-based monoliths, such as aerogels with honeycomb-like structure from a 2D material, in order to meet the requirements of TIMs with both high in-plane TC and through-plane TC, which sets them apart from horizontally or vertically oriented graphene materials with high anisotropy [21,22,23]. As well, we observed the applications of these 2D materials as phase-change materials with the development of graphene foam or aerogel as thermal switch materials, with the transition of graphene from thermally conductive to thermal insulating [24] and with the usage of graphene aerogels as a hybrid material with metals have also been under investigation [25].

The self-assembled hierarchical 3D filler networks with porosity can be fabricated as segregated or highly segregated structures containing large backfill space/voids in the matrix system. However, due to their large porosity, 3D monoliths’ TC are extremely low. Henceforth, in order to fill the porosity, pores are impregnated with thermally conductive polymers, with the aim to fill the air gaps with conductive long chains and construct thermally conductive pathways or channels inside for continuous phonon propagation and an increase in TC. Besides, this also provides higher mechanical stability without structure collapse.

## 2. Thermally Conductive 3D Aerogels Based on Carbon Nanofillers

Carbon nanofillers have sparked a lot of attention because of their unique features, including improved thermal and electrical conductivity, high mechanical strength, and ease of processing [26,27,28,29]. One viable technique for maximizing the benefits of carbon nanofillers is to segregate them into a strong three-dimensional (3D) structure for greatly improved properties relative to one-dimensional (1D) and two-dimensional (2D) structures. Such a 3D structure could now be attained by 3D frameworks/foams or aerogels-based composites or polymer-composite systems. Specific methods, such as chemical functionalization [30], orientation [31], preferential localization [32], and application of high-aspect-ratio nanofillers [33], have been developed to reduce thermal contact resistance accompanied by low filler fractions and excellent TC. Since thermal contact resistance is inversely related to contact area, filler thermal transfer efficiency should follow the rule of 0D < 1D < 2D < 3D, as the surface area grows with the filler dimensions (D). However, achieving high TC, while maintaining excellent electrical insulation performance, remains a significant challenge for these materials, due to the high electrical conductance of the 1D and 2D nanofiller particles. This section focuses on the TCs of carbon-based 3D framework structures composed of 1D and 2D nanofillers.

### 2.1. Thermally Conductive 3D Aerogels Based on 2D Carbon Nanofillers

**Figure 1:**
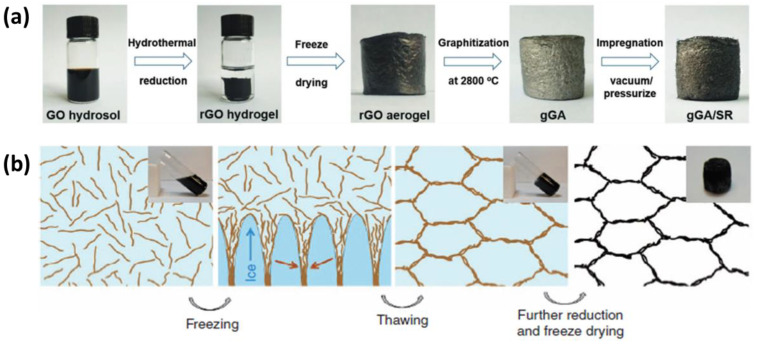
(**a**) Fabrication process of graphene/silicone-rubber-based thermally conductive aerogel. (Reprinted with permission from John Wiley and Sons, copyright 2019) [36]. (**b**) Formation of honey-comb-like structure (Reprinted with permission from nature communications, copyright 2012) [37].

In recent years, the most prevalent 3D aerogels are carbon-based structures with hierarchical anisotropic architectures, where the nanofiller is segregated into a 3D framework, elements of which are made up of interconnecting nanosheets or nanofibers, often aligned or oriented. This targets unique properties such as low density, high porosity, and super elasticity [34,35,36]. The self-organized 3D structuring is typically created by a phase-separation process, such as freeze drying, or by other means of segregation. The 3D architecture could be created as anisotropic, if the process is directed, for example, by thermal gradient. Other methods to introduce directed self-assembly process include application of an external force field or pressure, such as a gravitational, magnetic field, or electrical field, acting in a specified direction [37,38]. The structuring is followed by the self-orientation and aligning of 2D particles at the formation of walls of the resulting 3D architecture, e.g., by surface tension, van der Waals, or other forces. The result is a sort of highly directed graphene aerogel with a hierarchical structure and specific direction. The highly aligned graphene sheets might fully leverage their high intrinsic in-plane TC, generating a highly efficient thermal conduction network, as opposed to randomly scattered graphene sheets in isotropic aerogels. For this purpose of self-assembly, several techniques such as the freeze-drying method, followed by resin impregnation, have been used in recent years for the fabrication of vertically aligned graphene-based 3D networks [36,37,39]. For example, hydrothermal reduction, aligning graphene oxide (GO) nanosheets by freeze drying, and a subsequent 2800 °C graphitization were used by Wenya and coworkers [36] to create 3D graphene networks, impregnated later with silicone rubber (Figure 1a). Instead of van der Waals forces, they assumed the nearby graphene sheets in their work were joined by chemical covalent bonding, and, using vacuum impregnation and a high-pressure treatment process, graphene was thoroughly reduced, and the defects were totally eradicated. Meanwhile, prior studies on the graphitization of graphene mainly focused on removing the oxygen-containing functional groups from graphene and the use of van der Waals forces for the connection of adjacent graphene sheets [40]. For instance, a heat treatment process was used to create vertically aligned graphene hybrid foams/epoxy composites with a high out-of-plane TC, and the effects of the graphitization were studied [40]. The application of the graphitization is a very vital step to produce high-quality graphene, as it can eliminate most of the remaining oxygen functional groups in the reduced GO unit and correct defects. Hence, by following these steps, the out-of-plane thermal conductivity of the obtained thermal interface’s materials (TIMs) reached ~1.26 W/mK, when the graphene filler concentration was only 0.5 wt.%.

Qiu et al. [37] applied the freeze-casting method to create an interconnected graphene monolith with a honeycomb-like segregated structure, mimicking cork. The structure is unique for its kind and unprecedented, with the graphene foam exhibiting good flexibility, a light weight, and good mechanical strength rather than brittleness, without the need of infiltration by a polymer. The formation procedure of the cork-like monolith by freeze casting is depicted in a graphical illustration (see Figure 1b). When a well-distributed partially reduced graphene oxide (pr-GO) dispersion freezes, pr-GO sheets are condensed at the forming ice crystals’ boundaries and, subsequently, are aligned along the ice’s development direction due to the squeezing action; a continuous segregated honeycomb-like network emerges, as a result. When the ice is thawed, the framework particles remain inter-connected, preserving the structure. The findings suggest that such a robust structure could pave the way for the future scientific research of segregated carbonaceous fillers, to achieve good TC and other thermal properties. At present, various carbon-based aerogels with 2D nanofillers are being developed for numerous applications including wastewater treatment, energy storage and conversion, flame retardance, carbon dioxide capture, and catalyst supports and sensors; however, at the present time, they are now able to be employed to pertinent industrial applications, specifically related to TIMs.

**Figure 2:**
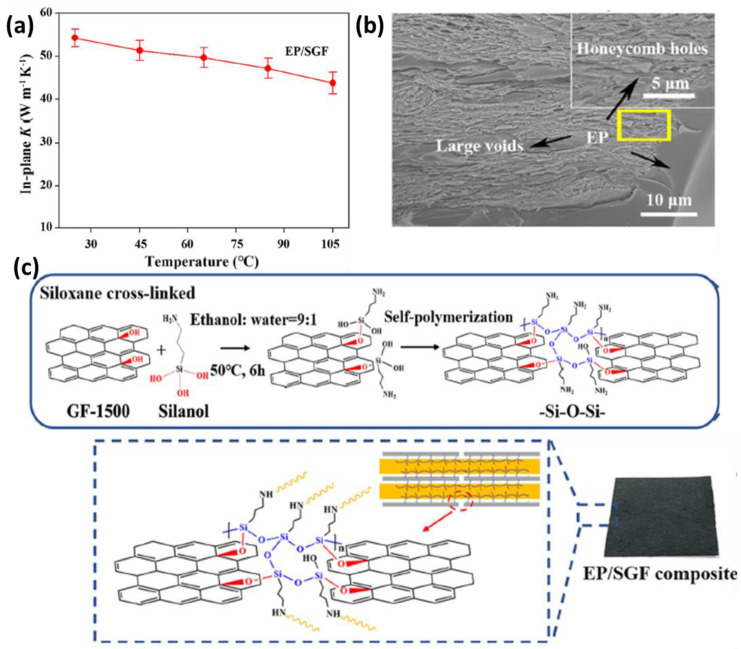
(**a**) In-plane thermal conductivity as a function of temperature for EP/SGF composite at a loading concentration of 20.2 wt.% graphene. (**b**) Cross-sectional SEM image of EP/SGF. The inset shows EP-filled honeycomb holes (Reprinted with permission from springer, copyright 2021) [52]. (**c**) Schematic diagram for synthesizing EP/SCF composites.

Various strategies for obtaining continuous 3D networks have been proposed in the literature [41,42]. In particular, substantial study has been done on the structure of graphene aerogels, and many studies on the production and engineering of aerogels with regard to the improvement of TC can be discovered [13,15,40,43,44,45,46,47,48,49,50,51]. Highly thermally conductive 3D structures were subsequently infiltrated with an epoxy resin to generate polymeric nanocomposites with increased phonons transport characteristics, due to the strong connectivity of graphene planes with the chains of polymers. Weng et al., for example, used a simple process to create highly thermally conductive paths: thermal-shock exfoliation of a graphene oxide sheet, followed by the self-polymerization of silanol inside graphene frameworks (GF), was executed [52]. The self-polymerization of silanol with graphene resulted in the surface modification of graphene by the silanol group. This chemical reaction further converted silanol into siloxane, with the cross-linking of graphene frameworks with siloxane networks (SGF) with a –Si–O–Si– molecular network structure formed by self-polymerization. The GF were then compressed, and the films, with tightly packed sheets, generated a microporous honeycomb structure with pore sizes varying from several to tens of micrometers, as shown in the SEM images in Figure 2. This porous structure was made up of horizontally aligned and locally attached graphene sheets, allowing it to function as a highly anisotropic, continuous network for phonon and electron transport. The epoxy (EP) resin was then infiltrated into GF to form the EP/SGF composite. After the impregnation by epoxy, a compression procedure was used on the GF sample to eradicate the voids and create a denser structure for enhanced TC, followed by thermal curing. Siloxane molecules, which were able to immobilize the graphene sheets in the epoxy matrix system, reduced the thermal resistances in the composite with the construction of siloxane molecular bridges inside the GF structure. Thus, the in-plane and through-plane TC values of EP/SGF composites with a GF loading concentration of 20.2 wt.% reached 54.2 and 1.62 W/mK, respectively, due to improved GF quality and significantly decreased intersheet and interfacial resistances. As another important consequence of the achieved results, we can state that these aerogels’ porosity can be altered, resulting in a different structural density and, consequently, in a variable amount of filler in the final 3D composite material.

The skeleton walls of the 3D graphene aerogels/foams used in thermally conductive polymer composites can be thought of as ultrathin graphene-based films. The quality of nanosheets and their aligned dense compaction are critical for heat conduction along the aerogel’s continuous skeletons. In reality, several approaches are developed to derive graphene aerogels/foams from GO sheets, and high temperature annealing is used to eliminate the GO sheets’ residual oxygen-containing groups and regenerate their lattices to heal defects in order to reduce phonon scattering [40,45,53]. However, the gases produced during the heat treatments and trapped at the skeleton walls may lead to thermally inert pores between the oriented graphene sheets, reducing the TC of the skeleton walls. For instance, Ruoff et al. used a chemical vapor deposition (CVD) technique to fabricate a three-dimensional (3D) graphene foam as the continuous thermal paths for the phase-change material. Nickel foam was employed as a template to deposit ultrathin graphite foam (UGF) with several graphene layers CVD-grown on the substrate, followed by the removal of nickel by the chemical etching process. The thin graphite hollow struts were then filled with hydrophobic paraffin wax, by immersing the foam in hot liquid wax. The fabricated composite of UGF/paraffin wax displayed the ideal phase change material, with a high TC of 3.44 W/mK at a very low graphene-loading concentration of 1.23 vol.% [54]. The TC increase can reach up to 1800%, relative to paraffin wax, as a matrix polymer with a specific TC enhancement of 1500% per 1 vol.% of graphene content. Thus, the construction of continuous graphene heat-transfer channels becomes an effective approach to improve the TCs of the polymer/graphene composite materials, due to the effective decreases in both the contact thermal resistance among the graphene sheets and the interface thermal resistance between the graphene and polymeric matrix [50,55].

**Figure 3:**
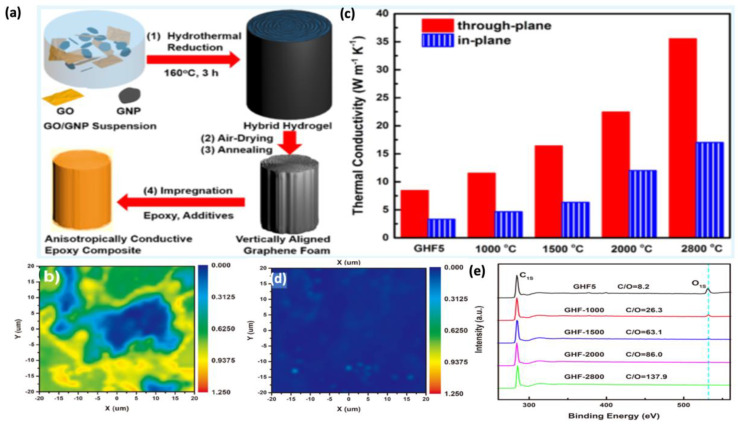
(**a**) Schematic illustration of the fabrication process: (**1**) vertically aligned reduced graphene oxide (RGO)/graphene nanoplatelets (GNP) hybrid hydrogels by hydrothermal process, (**2**) air-dried vertically aligned graphene hybrid form (GHF), (**3**) thermally annealed GHF, and (**4**) its epoxy composite. (**b**) Raman I_D_/I_G_ mapping of GHF5 (graphene oxide:graphene = 1:5) neat hybrid filler. (**c**) Effect of thermal annealing on the thermal conductivity of epoxy graphene hybrid foam composites conducted at various high temperatures. (**d**) Raman I_D_/I_G_ mapping of GHF-2800 (annealing at 2800 °C). (**e**) XPS spectra of annealed GHFs at different temperatures. (Reprinted with permission from American Chemical Society, copyright 2018) [40].

TIMs based on graphene offer a lot of potential for eliminating surplus heat created by electrical devices; however, their actual uses are frequently hampered by their poor TC, which is mostly caused by poor graphene-sheet dispersion and distribution. The TC-enhancement efficiency is still significantly below the theoretical value. The effectiveness of the 3D graphene network’s TC improvements remains a significant hurdle to attain a high through-plane TC of more than 10 W/mK, with a quite low graphene-loading concentration as a filler material in a polymeric matrix. Therefore, alternative approaches are constantly being tested for their efficiency. Fei et al. studied the hydrothermal reduction of reduced graphene oxide (rGO) in the presence of high-quality graphene nanoplatelets, followed by air drying and annealing, which created vertically aligned graphene hybrid foams (GHF) with high densities [40]. The resulting vertically aligned high-quality graphene porous structure with high density, as an ideal thermal conductance network of TIMs, is extremely effective in improving the TC of its composite; when impregnated with epoxy, it attains an extremely high through-plane thermal conductivity of 35.5 W/mK at a graphene loading of 19.0 vol.% (see Figure 3). The enhancement in the TC was mainly associated with an increase in the annealing temperature, in an ascending order ranging from 1000 °C till 2800 °C. The increasing trend of temperature exhibited the removal of defects inside the graphene structure, as observed in the Raman mapping and XPS spectrum, resulting in a higher TC. Raw (not annealed) GHF5 (the 5 comes from the mass ratio of graphene oxide:graphene = 1:5) and its thermally annealed equivalent GHF-2800 (GHF annealed at 2800 °C) are characterized using ID/IG Raman mapping (Figure 3b,d, respectively). A large blue region surrounds the yellow and green sections in GHF5 (Figure 3b). The blue regions correspond to high-quality GNPs with an I_D_/I_G_ value of less than 0.2, whereas the yellow and green regions, which have larger I_D_/I_G_ values >0.5, are caused by rGO sheets with residual oxygen functional groups and defects. GNPs are interconnected by rGO sheets, according to this I_D_/I_G_ distribution. The Raman mapping of GHF-2800, a GHF annealed at 2800 °C, is full of dark blue (Figure 3d), and most I_D_/I_G_ values remain below 0.025, because this temperature is high enough to entirely eliminate the leftover oxygen functional groups and repair the lattice defects. In support of these findings, XPS spectrum (Figure 3e) shows a decrease in the oxygen content, with a complete reduction in the oxygen peaks for GHF-2800. Hence, augmentation in TC of polymer composites can be directly linked to the annealing temperatures of their graphene structures for the removal of phonon-scattering defects and decreasing contact thermal resistance. The annealed graphene hybrid-foams-based epoxy composites are, thus, well-suited for TMA due to their outstanding TC characteristics, especially when the annealing is conducted at extremely high or ultrahigh temperatures, as supported by several authors [56,57]. Typically, graphene porous structures are made up of chemically or thermally reduced graphene sheets, which often have residual oxygen-containing groups and lattice defects that can produce phonon scattering [58]. The high-temperature annealing process, fortunately, has been shown to be effective in eliminating these functional groups and repairing the defects. For illustration, the preparation of thermally annealed defect-free graphene sheets was reported by Lian et al. [59], in which phonon-scattering centers and defects were greatly minimized, for the effective thermal transport of phonons by high temperature annealing, at around 2200 °C for thermally conductive phase-change materials, which displayed a high TC of 3.55 W/mK at a 10 wt.% of graphene-loading concentration. Similarly, in support of the aforementioned statements, Gao et al. employed a high-temperature annealing and mechanical pressing to create a high-quality graphene sheet with an ultrahigh TC of approximately 1940 W/mK and good flexibility for high-power electronic devices [60].

Most carbon-based aerogels have high intrinsic electrical conductivities, limiting their usage in electronic packaging materials’ systems due to issues such as short circuiting and other complications. As a result, converting carbon-based aerogels from electrically conductive to electrically insulating, while maintaining their outstanding mechanical and thermal qualities, may prove to be highly desirable and essential. Developing such carbon-based aerogels with a high electrical resistivity and heat dissipation capability, as well as strong structural stability, is now a major issue. Among several developed approaches, the nanohybridization of thermally conductive boron nitride with synergy with carbon-based materials such as graphene, graphene oxide, and reduced graphene oxide seems to be one of the most promising solutions to solve this problem [61,62,63]. For instance, the fabrication of 3D hybrid boron nitride graphene oxide nanosheets as aerogels with epoxy impregnation was explored, and the results showed an excellent TC of 4.53 W/mK (11.6 vol.% loading fraction) and good electrical insulation, with electrical conductivity reaching a low scale of 10^−11^ S/m, almost equaling the electrical conductivity of pure epoxy [63]. Moreover, for another different approach [13], a hydrothermal procedure aided by hydrofluoric acid produced highly compressible and thermally conductive, though electrically insulating, fluorinated graphene aerogels (FGAs). The introduction of fluorine to fluorinated graphene aerogels via the hydrothermal process results in a substantial reduction in charge-carrier density and changes the band gap of the inherent graphene. These aerogels have been shown to be very insulating, with the lowest electrical conductivity measured at 4 × 10^−9^ S/m, with the tunable band gap as reported in this work [13], which allows for the modification of their electrical insulating performance. Moreover, the electrical resistance of these FGAs varies depending on the deformation condition.

### 2.2. Thermally Conductive 3D Aerogels Based on 1D Carbon Nanofillers

Carbon nanotubes (CNTs) have been reported extensively for their usage as 1D carbon materials for 3D-based frameworks. Since their first discovery more than three decades ago, CNTs have drawn a lot of attention due to their exceptional physical, thermal, and mechanical properties [10,64,65]. In order to improve materials’ performance, CNTs are used as nanofillers in polymeric materials, such as epoxy resin, polyimide, and poly (methyl methacrylate), for achieving high TC. Due to their inherent high TC, of around 6600 W/mK (via MD simulation) and ~3000 W/mK (experimentally) [66], CNTs exhibit a vital role in TIMs for the dissipation of heat energy in electronic devices, with important factors including alignment/orientation, loading percentages, dispersion, density, aspect ratio, presence of topological and inherent structural defects, and chemical interaction of polymer matrix materials with CNTs [67,68]. In the case of a 3D framework aerogel structure [2,26], vertically aligned carbon nanotubes (VACNTs) are an ideal material for achieving a high TC with good mechanical characteristics [69]. By improving the contact at the interface or densifying the CNTs, the thermal interface resistance is decreased, resulting in an increased TC. Moreover, the heat transport can also be improved immensely by using high-temperature annealing to reduce defect density [70], utilizing mechanical densification to increase the volume percentage of CNTs [71], and enhancing the CNT alignment through processing with strong magnetic fields [72]. In thermally conductive aerogels with a polymer matrix, CNTs work in synergy with other carbon fillers to create a hybrid filler composite [65,73]. For instance, a unique class of paraffin-based shape-stable phase-change materials was fabricated by vacuum-assisted impregnation of paraffin into an aerogel comprised of reduced graphene oxide (rGO) and carbon nanotubes (CNTs). Investigations were done into how the morphology, structure, and properties of the paraffin composite were affected by the ratio of rGO to CNTs in the 3D-network structure. The 3D-network topology of rGO/CNTs possessed a high TC and acted as a single thermally conductive skeleton with good stability. 

**Figure 4:**
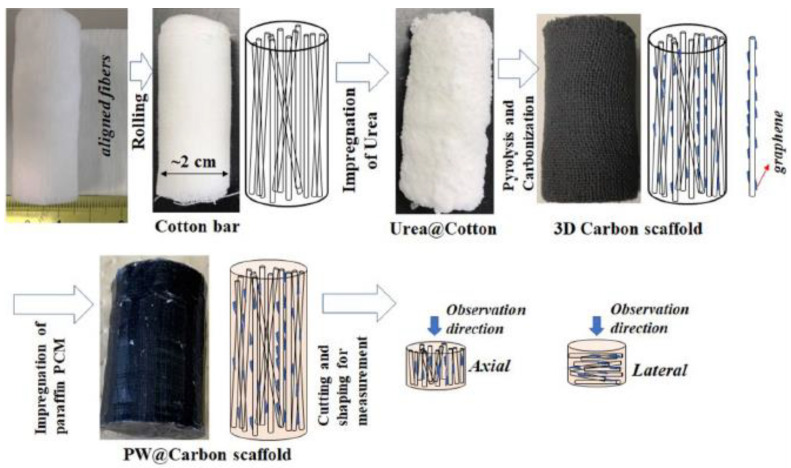
Schematic for the fabrication of 3D carbon-fiber-wrapped graphene phase-change composite (Reprinted with permission from Elsevier, copyright 2022) [78].

Other than carbon nanotubes, carbon fibers have been used as 1D fillers in 3D polymer composites for TMAs [74,75,76,77,78]. These carbon fiber composites are typically fabricated by the combination of several steps, including carbonization, graphitization, and high-temperature hot-press molding techniques, and possess high TCs in both the axial and radial directions, with in-plane TC exceeding 900 W/mK [79,80]. Little work has been done on the thermally conductive 3D-based aerogels based on carbon fibers, but, nevertheless, 3D-based fabricated structures of 1D carbon fiber have been reported with increased interest, in recent years. For instance, in their experimental study, Yao et al. first created porous carbon-fiber (C/C) composites using low-temperature hot-press molding [81]. Following that, a chemical vapor infiltration (CVI) technique, precursor impregnation, and pyrolysis were used to densify the porous C/C composites. Finally, carbon bulks with a density of 1.90 g cm^3^ and in-plane TC of 667 W/mK were produced, following the graphitization process at 3000 °C. Similarly, in a study conducted by Zhang et al., the researchers densified large-diameter graphite fiber bundles and developed a method to synthesize two types of carbon rods without the use of hot pressing [74]. One kind was fabricated by injecting phenolic resin into the bundles of carbon fiber, followed by curing, carbonization, graphitization, and densification with a CVI technique, while another type was created by subjecting the carbon bundles to CVI treatment, with further densification of both types of carbon rods finally. After graphitization, these kinds of carbon rods achieved TCs of around 569 and 675 W/mK, respectively. Moreover, in another study conducted by Nan Sheng et.al, the role of wrapped graphene on the vertically aligned hollow carbon fiber was investigated with impregnation by Paraffin wax [78]. Biomass cotton was used to fabricate porous carbon scaffolds as the supporting framework for creating high-performance phase-change materials (PCMs), in order to address the issues of the leakage and poor heat conductivity of the composite materials based on paraffin (Figure 4). The phase-change composite (PCC) with an 8.5 wt% filler concentration demonstrated a high TC along the axial fiber direction of 2.68 W/mK, which is 10 times greater than that of paraffin. The segregated carbon fibers are connected by the porous carbon frameworks produced by the introduction of urea, which are tightly coiled around the fibers and filled in where there are gaps. Hollow carbon fibers and porous graphene frameworks are part of a three-dimensionally connected and crosslinked carbon network that can provide various, continuous pathways for the thermal-conduction phenomenon. Particularly, the high TC of graphene type carbon is projected to significantly improve the PCC’s ability to conduct heat and to be used in applications related to TMA.

**Table 1:** Thermal conductivities of various aerogels 3D frameworks, as mentioned in the literature.

Polymer Matrix	Filler Loading	Type of 3D Structure	Thermal Conductivity, W/mK (Through-Plane)	Thermal Conductivity, W/mK (In-Plane)	Technique for Measuring Thermal Conductivity	References
Epoxy	25.4 vol.% graphene/boron nitride	Graphene/boron nitride aerogel	11.01	5.9	Laser flash	[61]
Epoxy	2.5 vol.% graphene	Graphene aerogel	9.1	3.6	Laser flash	[61]
Epoxy	19.0 vol.% graphene oxide/graphene nanoplatelets	Vertically aligned graphene hybrid foam	35.5	17	Laser flash	[40]
Polydimethylsi- Loxane (PDMM)	11.62 wt.% graphene	Graphene foam	1.62	28.77	Laser flash	[50]
Epoxy	0.75 vol.% graphene	Graphene aerogel	6.57	1.1	Laser flash	[49]
Epoxy	2.30 vol.% graphene	Polyamic acid salt/graphene oxide (PAAS/GO) hybrid aerogels	20	17	Laser flash	[48]
-----	Density of graphene aerogel 150.49 mg cm^−3^	Graphene aerogels films	0.7	53.56	Laser flash	[47]
Paraffin wax	----------	Polyamic acid/graphene oxide aerogel	2.68	8.87	Laser flash	[46]
---------------	5 wt.% graphene	Graphene aerogels	----------	4.28	Laser flash	[45]
Epoxy	7.2 wt.% (6.0–1.2 wt% copper– graphene)	Copper nanowires/graphene aerogels	------------	0.51	Hot disk	[44]
Epoxy	-----------	Fluorinated graphene aerogel	------------	2.5	Steady-state method	[13]
---------	100 wt.% boron nitride nanosheets	Boron nitride aerogels	-------------	25.2	---------	[82]
Polyethylene glycol	-----------	Boron nitride aerogels	-----------	0.67	Laser flash	[83]
Poly vinyl alcohol	0.12 wt.% boron nitride nanosheets	Boron nitride/polyvinyl alcohol hydrogels	------------	0.68	Transient plane source method	[84]
Poly vinyl alcohol	67.7 wt.% boron nitride	Boron nitride/polyvinyl alcohol aerogels	1.1	10.04	Laser flash	[85]
Cellulose nanofiber	50% wt.% boron nitride	Boron nitride (BN)/cellulose nanofiber aerogels	------	2.71	Laser flash	[1]
Polydimethylsiloxane (PDMS)	3.32 wt.% reduced graphene oxide (rGO)-cellulose	Reduced graphene oxide (rGO)/cellulose polydimethylsiloxane	-------	0.65	Laser flash	[86]
Cellulose	33wt.% boron nitride/cellulose	Boron nitride–nanosheet/cellulose nanofiber aerogel	-------	0.57	Hot wire transient method	[87]
Epoxy	34 vol.% boron nitride	3D boron nitride aerogel/epoxy	------	4.42	Laser flash	[88]
Epoxy	9.6 wt.% boron nitride	3D carbonized cellulose aerogel/epoxy	-------	2.11	Hot disk	[89]
Parrafin wax	1.23 vol.% graphene	Ultrathin graphite foams	-----------	3.44	Steady-state method	[54]
Epoxy	1.0 wt.% MXene (Ti_3_C_2_)	MXene (Ti_3_C_2_)/epoxy	-----------	0.587	Laser flash	[17]
Polydimethylsiloxane (PDMS)	25.4 wt.% boron nitride	Boron nitride/PDMS	---------	1.5	Laser flash	[90]
Polyvinyl alcohol	66 wt.% boron nitride	BN/PVA aerogel cake composite	0.61	0.76	Laser optical thermal scanner	[38]

## 3. Cellulose Based Thermally Conductive Aerogels

Cellulose, with an intrinsic TC that is ~0.04 W/mK, is a biologically friendly compound that is rich in oxygen and hydroxyl functional groups, which shows good compatibility with most of the 2D-based nanofillers, for the formation of thermally conductive aerogels [86,87,89]. One such thermally conductive fillers utilized in cellulose-based composites is boron nitride (BN) (also known as white graphene) [91]. BN can be exfoliated into boron nitride nanosheets (BNNS), which are 2D nanosheet structures with strong thermal conductance characteristics [92,93]. BNNs, featuring a graphene-like crystal structure, not only offer outstanding mechanical stability and thermal conductivity (~600–2000 W/mK) but also have low coefficient of thermal expansion, good electrical insulation, antioxidative properties, and thermal stability [94,95,96].

**Figure 5:**
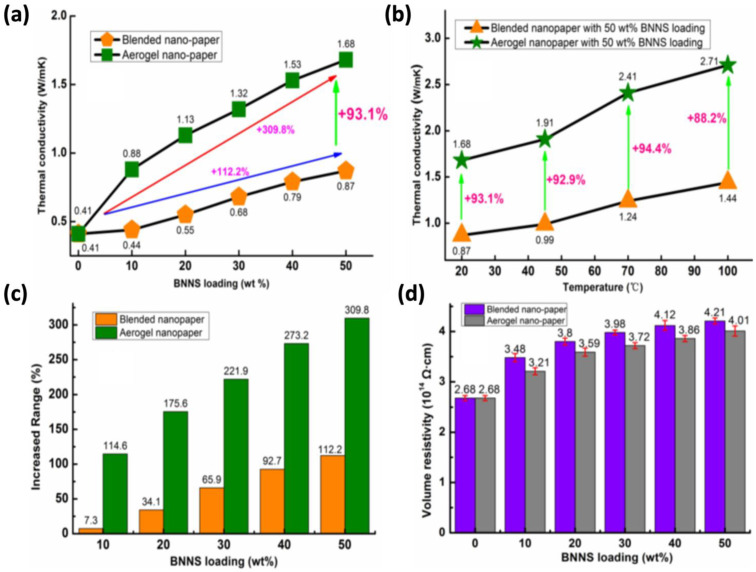
Blended nanopaper vs. aerogel nanopaper: (**a**) thermal conductivities with different BN loading content; (**b**) thermal conductivities at elevated temperatures with BN loading content of 50wt.%; (**c**) aerogels’ composite enhancement factor; (**d**) electrical volume resistivity [1].

The development of a 3D-interconnected network structure based on BNNs can improve the thermal transfer efficiency per unit mass, i.e., TC, for lightweight, portable electronic equipment. This material can be employed in 3D aerogels for a variety of applications, including insulation and fire protection, as a host for polymer matrix composites, and so on. In order to enhance the TC of BN aerogels, Wang et al. [86] fabricated boron nitride (BN)/cellulose nanofiber (CNF) composite structures. They varied and adjusted the BNNs content in the CNF and made nanopaper using two methods: simple blending and the aerogel 3D-skeleton approach. The aerogel-based nanopaper had 93.1% better TC (2.71 W/mK) than the blended nanopaper (1.44 W/mK) with 50 wt% BNNs loading, as shown in Figure 5a. The higher TC of the aerogels could be owing to the BN nanosheets spreading more evenly and uniformly inside the cellulose matrix compared to the paper-blending process. Furthermore, compressing the porous aerogels reduces the amount of trapped air bubbles/pores, allowing the boron nitride nanosheet contacts to be closer together, for better phonon transfer inside the composite system. The volume electrical resistivity of the BNNs/CNF nanopaper (3.8 × 10^14^ Ω cm) (Figure 5d) was also found to be higher than that of pure CNF (2.6 × 10^14^ Ω cm), which is still much higher than the insulation requirement (electrical resistivity > 10^9^ Ω cm) [97]. Besides, the aerogels demonstrated qualities such as high strength, low thermal expansion coefficient, and environmental friendliness.

**Figure 6:**
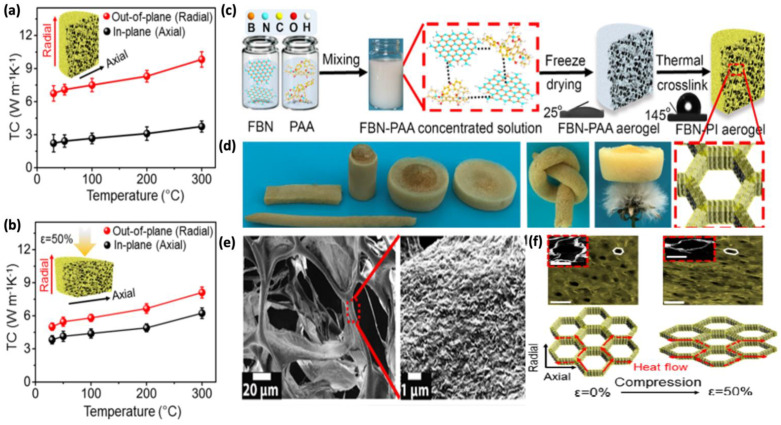
Thermal performances of aerogels: (**a**) thermal conductivities of FBN-PI aerogel without compression at different temperatures; (**b**) thermal conductivities of FBN-PI aerogel with 50% compression at different temperatures; (**c**) schematic illustration of the fabrication process of FBN-PI aerogel; (**d**) digital photographs of FBN-PI aerogels with different shapes, under knot, and floating on the flower stamen; (**e**) SEM and TEM images with different magnification of FBN-PI aerogels; (**f**) optical microscope images (200 μm) and SEM images (50 μm) of FBN-PI-2 aerogel from 0% strain to 50% strain and corresponding anisotropic thermal conduction mechanism [98]. (Reprinted with permission from American Chemical Society, copyright 2019).

The 3D aerogels synthesized from hydroxyl functionalized boron nitride nanosheets (BNNs) via the freeze-casting method can have a high TC over a wide temperature range. As the temperature tends to increase from 30 °C to 300 °C, the in-plane TC of functionalized BN-polyimide (FBN-PI) aerogels with 50 wt.% filler loading increases from 6.7 W/mK to 9.8 W/mK, with an ultralow density of 6.5 mg/cm^3^, as shown in Figure 6a [98]. This enhanced TC is highly suited for high temperature TMA, especially as a lightweight thermal conductor.

In the condition where the same aerogel is compressed, the TC increases only moderately as the temperature rises. Despite Umklapp phonon-scattering, this phenomenon results from the rising gas’ thermal radiation at elevating temperatures. Under compression, the out-of-plane TC decreases significantly, while the in-plane TC rises, owing to a change in the anisotropic cellular honeycomb structure, with increased axial heat transfer extension with stretched boron nitride bonds (see Figure 6f). Furthermore, after compression, the reduced air gaps between the layers aid in the improvement of the in-plane TC. The honeycomb cell is elongated parallel to the radial direction (out-of-plane) under a compression deformation of around 50%, resembling the layer-by-layer structure. As a result of this, the in-plane TC increases, while the out-of-plane TC decreases, causing the TC to shift from thermal anisotropy toward isotropy. As a result, it is possible that the elastic thermal anisotropic property of the FBN-PI aerogel could be used for thermal-based strain sensors, and, furthermore, this proves that BNNs-based aerogels may be used to manage thermal energy even at high temperatures.

**Figure 7:**
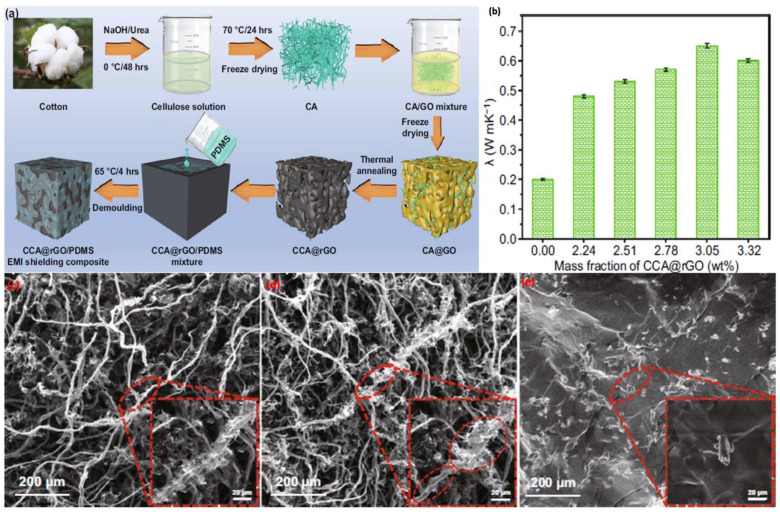
(**a**) Schematic illustration of the fabrication procedure for rGO-cellulose/PDMS aerogels; (**b**) thermal conductivity of rGO-cellulose/PDMS aerogels composites with various filler loading of rGO-cellulose in PDMS as matrix [86]; (**c**) SEM images of CA; (**d**) CCA; (**e**) CCA@rGO-based PDMS composites.

It is not necessarily required to create 3D-framework filler/polymer in one manufacturing step; a polymer framework can be filled with filler material afterward, providing suitable thermal properties. In one of the findings reported recently, reduced graphene oxide (rGO)/cellulose-based aerogels were fabricated via a two-stage freeze drying and self-assembly technique, where first the structure of the cellulose aerogels (CA) was formed, followed by a second step, freeze drying it immersed in GO as a filler mixture [86]. The mechanism includes the gradual wrapping of rGO on cellulose fibers, for the formation of a 3D double-layered thermally conductive network with a skin-core-type structure. After annealing, the single nanofibers of carbon cellulose aerogels (CCA) have a twisted-like structure and are around 6 µm in diameter, which is mostly due to the removal of oxygen-containing functional groups and the carbonization of the cellulose (see Figure 7c–e). The annealing of aerogels was executed, followed by infiltration with polydimethylsiloxane (PDMS) via the vacuum impregnation technique, to form the final composite. The skin-core structure of CCA@rGO is substantially conserved after backfilling with PDMS, and the 3D double-layered thermally conductive network structure of CCA@rGO is not considerably degraded, with the PDMS disseminated more uniformly in the gaps of the 3D thermally conductive network of CCA@rGO. As a result, the composite exhibited enhanced thermal conductivity of 0.65 W/mK, with an excellent electromagnetic shielding effect.

### Biomass-Based Carbonized Cellulose Thermally Conductive Aerogels

**Figure 8:**
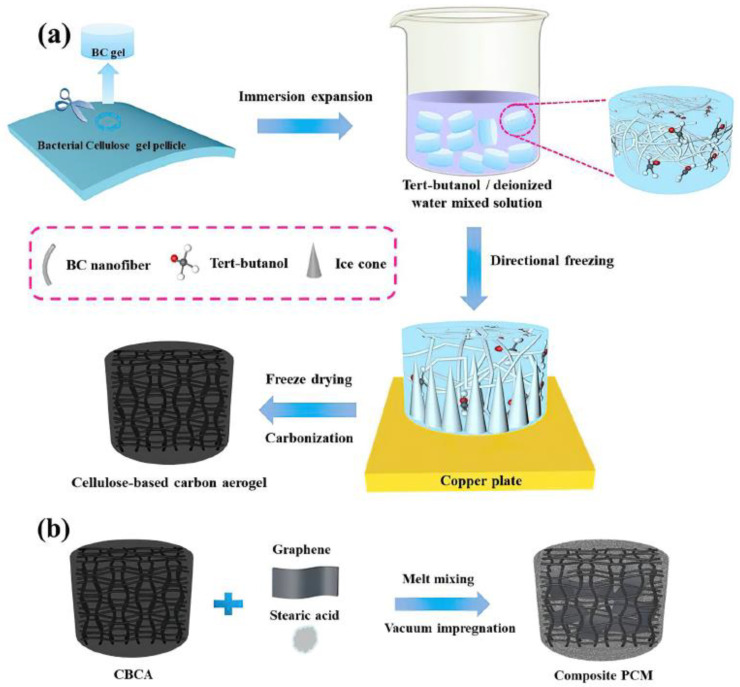
Schematics of the fabrication of: (**a**) cellulose-based biomass carbon aerogel preparation and; (**b**) composite phase-change material. (Reprinted with permission from Elsevier, copyright 2022) [101].

Due to their abundance of supplies, affordability, environmental friendliness, and naturally porous nature, biomass materials are widely desired for solar applications. One of the most alluring ways to increase the stability and effectiveness of solar energy is to use energy-storage strategies based on organic composite with a high TC and strong shape stability. With the improvement of the TC and shape stability of organic composites, they are reported to be achievable through the creation of three-dimensional heat transfer scaffolds [99]. For this purpose, cellulose-based carbonized aerogels are employed [100,101,102]. Wu et al. reported a biomass-carbonized aerogel with a TC of 1.17 W/mK, where cellulose was selected as a precursor for the carbon source [101]. Techniques involving immersion expansion, orientation, freeze-drying, and carbonization methods were tested to create 3D cellulose-based carbon aerogels. After carbonization, the procedure involved the impregnation of various volume and weight percentage concentrations of stearic acid/graphene mixtures, by vacuuming them for more than 12 h to completely remove the trapped air bubbles (Figure 8). The aerogel exhibited a high specific surface and productivity as well as minimal structural shrinkage, when tert-butanol/deionized water was used as the cosolvent. The use of graphene and a 3D-directed graphitized porous network ensured good broadband absorption, effective heat transport, and efficient thermal storage.

## 4. Carbon Nanomaterial-Based In Situ Construction of Thermally Conductive 3D Frameworks with Polymers

**Figure 9:**
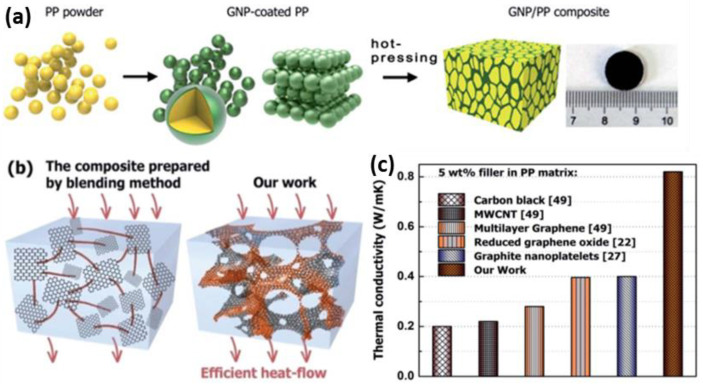
Schematics of the fabrication process of GNP/PP framework composites. **(a)** Fabrication of GNP-coated / PP composites; (**b**) Diagram comparing effective thermal conduction through a network of connected graphene to that through dispersed graphene; (**c**) Comparison between the TC values of these composites and randomly dispersed filler composites with the TC of GNP/PP composites (Reprinted with permission from Royal Society of Chemistry, copyright 2017) [106].

The ability of polymers to transmit heat must be improved by the creation of an interconnected network. Adding prefabricated 3D networks as fillers is a successful way to create continuous heat conduction networks in polymer matrices. However, other approaches, including adding a higher weight percentage of particle fillers, are less effective, as they deteriorate the mechanical integrity of thermally conductive polymer composites. However, the in-situ fabrication of a connected conducting composite network is another easy and scalable method that has been reported in recent years for fabricating thermally conductive 3D-framework polymer composites [103,104,105]. In this procedure, following the fabrication of carbon nanomaterial-based polymer composites, polymer powders are first precoated with a thermally conductive filler before being hot-pressed and compressed into a monolith framework. For instance, graphene/polymer composites were fabricated by Lin’s group, by coating graphene on polymer powder first, then drying and hot-pressing it to form a 3D scaffold structure [106]. In light of this, a continuous graphene-conducting polymer matrix network is established, and this method can be employed with most of the thermoplastic polymers, including polyethylene, polypropylene (PP), polyvinyl alcohol (PVA), and polyvinylidene. Hence, as shown in Figure 9, the TC of PP composites with graphene network connections, built in situ, was far more substantial than those of composite materials with randomly displaced/positioned graphene in a PP polymer. Using this similar method, Wang’s team fabricated and developed a highly organized 3D CNTs/polystyrene composites framework for a high TC [107]. After compression and heat-treatment processes, a 3D CNT interconnected structure was created in the polystyrene matrix by coating the polystyrene (PS) particles with MWCNTs. With the help of this procedure, distinctive composite structures with superior properties were formed, and the composites displayed a continuous phase structure of the PS phase and MWCNT network by incorporating an intact, uniform, and well-defined 3D network of MWCNTs.

Apart from such benefits, however, this strategy has a number of drawbacks. First, only thermoplastic polymer materials may be prepared using this thermoforming technique, so it is frequently necessary to process the polymer into tiny particles beforehand [106]. Second, the formation of an anisotropic, thermally conductive networks composite is a serious challenge with this technique, as it favors the formation of isotropic networks with a comparatively low TC, with respect to the high TC reported in recent years. As a result, the applicability of this in situ construction method for 3D interconnected conducting network is limited.

Table 1 shows the thermal conductivities of various 3D aerogels, with the reference to the testing procedure involved. It demonstrates the significant quantity of study being conducted globally to determine the thermal conductivities of 3D aerogels frameworks.

## 5. Conclusions

This brief study provides an overview of the literature on the thermally conductive three-dimensional frameworks that have been developed in recent years, for their use in thermal management applications. We expect that, in the near future, these thermally conductive frameworks will yield some significant findings for their applications in thermal management, particularly as thermal interface materials, phase-change materials, and thermally conductive structures such as aerogels/foams/sponges, which are expected to reshape the electronics industry. Most researchers have been working on constructing highly porous 3D-skeleton structures, with aligned thermally conductive fillers such as graphene and boron nitride, in recent years. These porous microstructures aided in the infiltration and intercalation of the inserted polymer chains, for the bridging effect of an effective phonon transfer, resulting in the increased thermal conductivity of the whole 3D framework. Furthermore, the filler thermal transfer efficiency of 3D structures results in a larger effective contact area with the impregnating polymer chains, lowering the composite system’s thermal resistance. Further investigation into thermally conductive 3D structures could be very promising in the electronics sector, since it could lead to the development of low-cost, highly thermally conductive components that are easier to fabricate and scale up than conventional phase-change and thermal interface materials. The high electrical insulative properties accompanying the desired thermal conductivity values present a challenge, even nowadays, highlighting one of the trends for future endeavors.
